# Barriers of Developing Medical Tourism in a Destination: A Case of South Korea

**Published:** 2017-07

**Authors:** Ladan ROKNI, Turgay AVCI, Sam Hun PARK

**Affiliations:** 1. Dept. of Tourism Management, Eastern Mediterranean University, Famagusta, North Cyprus; 2. Asia Content Institute, Konkuk University, Seoul, South Korea

**Keywords:** Medical tourism, Cultural competence, Linguistic proficiency, International healthcare, Korea

## Abstract

**Background:**

This study aimed to determine the efficient factors that potentially lead to the barriers of developing medical tourism in South Korea.

**Methods:**

To explore the current medical tourism trend, a qualitative procedure was adopted. Besides analyzing the current situation of medical tourism in Korea through a systematic searching on the available information and publications, in-depth-interviews were conducted to collect data from relevant authorities and representatives of medical tourism associations in this country.

**Results:**

The result revealed, although government have supported this industry, that lack of specialty and expertise among the health care practitioners in the scope of cross cultural communication, seems to be the core barrier to development of medical tourism in Korea. Demands for convenient promotional activities, policy making and action regulation are the other effective factors.

**Discussion:**

Several strategies are required in order to address and combat these barriers, such as governmental support for cultural training, cooperative efforts to encourage health practitioners involved to enhance their cultural and linguistic competence in international scale.

## Introduction

Medical tourism has emerged as a new type of tourism and healthcare service mobility (1, 2) since it demands for a service and arrangement, which is beyond the current regulations of both tourism and healthcare sectors. Acknowledging that, as for medical tourism people tend to travel beyond the national borders demanding for a medical-health service, a new type of mix service would be required. The demand side in such a definition are named neither tourist nor patients (3), but patient-customers (4). These situation leads to a variety of ambiguities as to whether medical tourism could be developed through tourism strategies, medical excellences or even other external factors. Nevertheless, the role of supply side seems to be exponentially essential, which includes a chain of providers. The issue that what organization(s) is/are/should be included in offering medical tourism service is the matter of dispute which could potentially lead to inconsistency and barriers in the way of development.

Despite the remarkable contribution of medical tourism in the country’s economy (5, 6), it does not seem easy to catch that place. There are pivotal factors that play an essential role in order to lead the countries to be beneficial financially; for instance, the importance of providing particular quid lines and certification procedures have been highlighted (7), establishing a patient oriented service system (8) or even a marketing promotion system (9). Moreover, the contribution of countries in this globalized and competitive arena requires an adequate response of both tourism services and infrastructures (1) and collaboration of private facilitators and the entire country (10).

In many Asian countries, as the main destinations for medical tourist, governments have invested into infrastructure and promotion of medical tourism (11). Thailand increased its international reputation by providing a very cozy atmosphere for foreign patients by giving the appearance of high class hotels to its hospitals, also more important by establishing constructive relationships of patients-doctors (12). The situation in Malaysia, though, owes to its cultural diversity which has provided a competitive advantage through cultural competence (4); since the mixture of Malay, Chinese, Indians and indigenous Ethnic groups, resident in Malaysia, caused the attraction of patients from major civilizations in Asia (13).

The market environment of competition in Asia, lately, influenced on the growth rate of foreign patients coming to Korea. This country has faced a complex situation in which although many essential factors are available, in a high class and quality, lately the number of foreign patient has decreased (14). Acknowledging the current movement in the region, the situation of medical tourism in Korea demands for a revision on the effective factors that might contributes to leading the country in a way to be competitive again.

Accordingly, the aim of this study was to investigate and explain the effective factors that influence the current trend of medical tourism of Korea. Particularly we aimed to inquire the lack of qualifications, which function as the barriers to medical tourism development in this country. The finding will contributes to the literature by clarifying the barriers in medical tourism and can be generalized and applied to countries with similar situation; also the conclusion might practically be noticed by the authorities in order to address the current shortages.

### South Korea

South Korea has made effort continually to strengthen its medical tourism by applying IT knowledge to design the facilities and hospitals, also marketing its IT-medicine beyond the borders (15–17). Among 30 medical tourism destinations, this country ranked 17^th^ based on a variety of criteria (18).

Officially medical tourism service has been offered since 2009, after passing 4 years of strategic planning and clarifications in terms of the requirements and legislation, starting from 2005 and by the governmental support over this period (8). By 2014, meanwhile, the country experienced a significant increase in the number of foreign patients. In fact, by 34.7% increase annually, the figure for arrival foreign patients soared from 60,201 in 2009, to 266,501 in 2014 (14).

While the private sectors are highly active, governmental organizations and committees support them systematically. Stablished committees are: ‘Council for Korea Medicine Overseas Promotions (CKMOP)’ to lead the ‘communication activities with international patients’ (19), Korea health industry development institute (KHIDI), ‘The committee for an advanced medical industry’, Korean international medical association (KIMA), Korean tourism organization (KTO), Korean Institute for healthcare accreditation (KOLHA), etc., Beside the committees, is the financial support and investment that government put aside for marketing activities and infrastructure, respectively (14). Stablishing ‘Medical Tourism Visa’ and evaluating hospitals regularly, are the other major activities of Korean government. Many hospitals, clinics and coordinator have been registered according to the evaluation standards designed by government, to provide healthcare services to foreign patients (20).

Despite designing such an accurate system, South Korea lately faced not a considerable increase in number of foreign patients, comparing to its competitors in East-Asia regain. Government has invested 400 million US dollar annually (14), while this amount of money is far more than the amount turned back. So far, a range of factors have been mentioned as the root cause of this new trend, for instance insufficient customer service quality (15), limited and weak exchange of information, lack of a patient-oriented service system (8); nevertheless, still the main contributor(s) to this problem is a matter of dispute. Undoubtedly, what is bold in Korea, is high quality of medicine, both procedure and facilities; but in order to provide a sustain development, Medical tourism in Korea demands for a comprehensive revision.

### Model

Yet, there are absolutely valid arguments on the issue of involved factors on development of medical tourism industry, but lack of consensus can be seen among them. By the way, the influence and requirements of medical tourism, in a national scale, have been conducted by several studies (21–23). Meanwhile, these models/frameworks are mostly focused on the patient’s decision making, the components of the medical tourism market, pull/push factors and motivation theories. Only one single framework focuses on the influential factors to develop medical tourism in a country (24). This conceptual model has two parts –supply and demand side- which represents the comprehensive image of medical tourism requirements in a country, and was adopted here.

In our study, similar to a research conducted in Hong Kong (25), we just considered the supply side to investigate how well Korea is prepared to address the requirements of medical tourism in order to reach to its barriers. Applying this model enabled us to have an initial framework for gathering information and conducting the interviews in an appropriate path as well.

## Methods

**Fig. 1:**
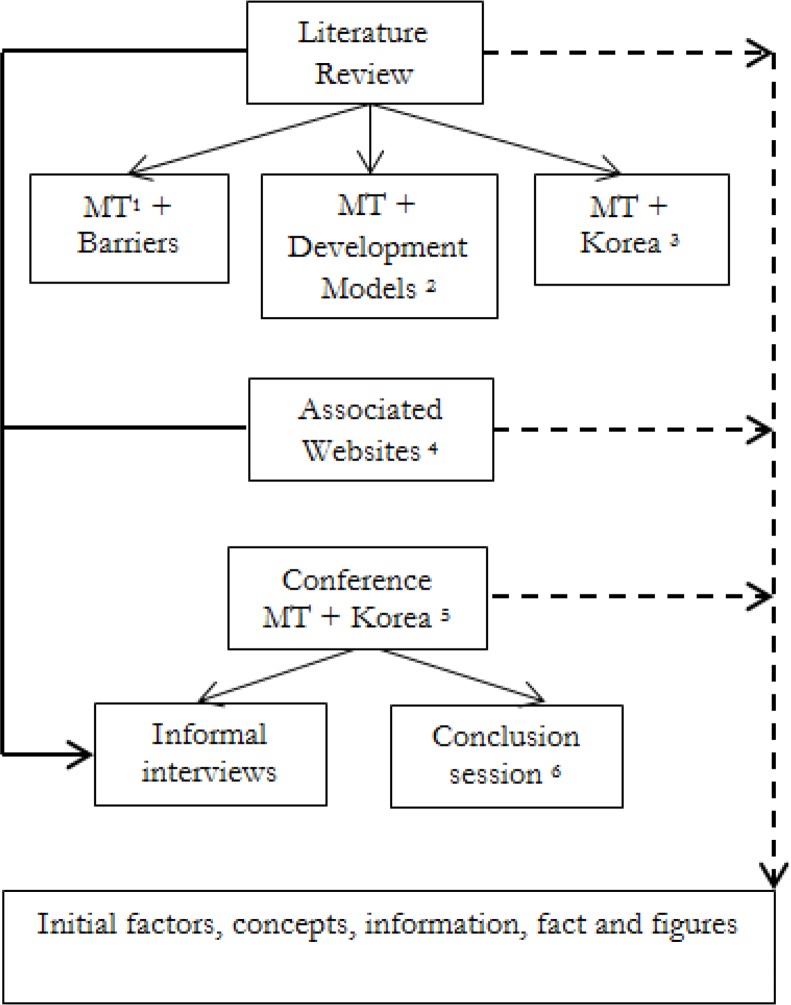
Summary of the review process

An exploratory approach through a qualitative research method was adopted since the initial information was required to lead the authors to identify the variables for their further research (26). Extensive and systematic reviews were performed on the available information, as shown in Fig. 1 several steps were taken to achieve the valid information in order to explore the characteristic of current medical tourism trend in Korea and its barriers to development.

Identifying the key issues enabled the authors to have a list of initial factors which could potentially contribute to the barriers and shortages of Korea to develop medical tourism. Next, the relevant authorities and experts -15 individual- were interviewed to confirm or modify the factors trough an in-depth and open-ended questions. During the interview, researchers tend to discuss the medical tourism trend in Korea, rather than asking a pre-framed question and expecting a specific response. This method provided more explorative-oriented information comparing to semi-structured interviews (27). The interviewees were all directly involved in medical tourism of Korea and were either representative of relevant authorities in medical and tourism sectors, or active experts researching on Korean medical tourism.

1. Medical Tourism
2. Includes the published papers regarding medical tourism development models and requirements
3. Includes published papers regarding medical tourism in Korea
4. The available information within the website of organizations in Korea associated with medical tourism industry
5. The conference of Medical Korea and K-Hospital.
6. “Global healthcare policy and management forum”. This session was held with the aim of discussing the main shortages of Korea in terms of medical tourism and based on the current trend in other countries.

### Data analysis

Having all the clear information about the current situation of medical tourism in Korea, besides the valid opinion of relevant authorities in the scope of shortages, “content analysis” was undertaken on the gathered information; following are the particular stages of interpretation. All the information was reviewed for identification of any “data redundancy” in the format of concepts, phrases or words; accordingly, themes and sub-categories emerged. The main categories, refinement and identifying the relationship among them were manipulated by employing “Axial coding” (28). Finally, the information was compared with the secondary documents and the information gathered by observations (29). For developing the framework that represents the relationship among the factors, we adopted the procedure of (25), who presented the barriers to medical tourism development in Hong Kong based on a model, namely, “supply and demand of medical tourism” (24).

## Results

Data were collected through an extensive literature review and in-depth interviews. Since the interviewees were all informed of general/current framework in terms of medical tourism in Korea, we considered their notions as the valid and potential obstacles to development. Noteworthy is the fact that their notion was a confirmation on the initial pool of barrier’s concepts collected from literature.

**Fig. 2:**
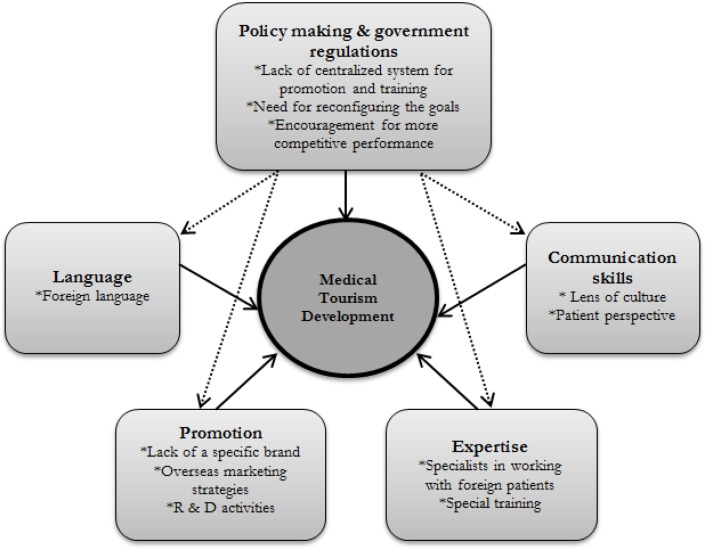
Framework of barriers to medical tourism development in South Korea

Among variety of ideas those with a high range of redundancy were revealed, also finding the similarity with the available and pre-published information improved the validity of developed framework, presented in Fig. 2.

As shown, five main themes were selected as the barriers to medical tourism development in South Korea; each theme entails a number of categories which could participate in leading to the associated theme. This framework developed based on the “amalgamation” of the achieved themes and categories and the arrows illustrate the relationships among them. Based on the current situation, all the themes has the direct influence on the development of medical tourism in Korea, shown by solid arrows, namely, policy making and government regulations, expertise, promotion, communication skills and language. Meanwhile, dashed arrows represent the interrelationship among these themes; also particularly the key influence of “policy making and government regulations” on the other themes. For instance, in terms of addressing the problem of trained specialist in medical tourism, the policy making and expertise were found to have overlap.

While it was widely accepted that a correct policy making and reconfiguring the goal could provide a positive development, providing a communication, from “patients’ own cultural perspective” was cited as the chief obstacle to the development of medical tourism in Korea, with the most redundancy level among the factors.

## Discussion

Based on the gathered information, the framework was developed in order to identify the problems and obstacles that effect on development of medical tourism in South Korea. Comparing with previously stablished models, also suggested barriers in other countries and Korea; many similarities can be seen, especially in terms of general policies and communication skills. Nevertheless, there are some differences to pre-identified factors (21, 22) owing to the high quality facilities and medicine in Korea, which has been already, established.

Some of the barriers identified in this study, are similar to those in Hong Kong (25), such as promotion, expertise, policies, communication, and language. Moreover, similar to our study, the lack of regulation and training, also lack of “physician expertise” has been identified as the barriers in a general development model of medical tourism (22). Particularly, in terms of Korea some factors have been confirmed so far, such as, lack of insufficient promotion, centralized administrative support system and medical tourism professionals (8) also the need for a revision on the performance of medical staff’s manner (17). The case study carried out by Jun (2016) on the general characteristic of Korean medical tourism, identified some factors similar to those revealed here, for example “English language service” and “specializing in foreign patient care”, with 94 and 50 % repetition level, respectively (15).

The chief barrier, among the themes was “communication skills”. It implies on the effective and appropriate cultural competence that enables the health practitioners to avoid any generalization and offer a cultural service particularly for each patient with different cultural background and personal background as well. In terms of developing medical tourism in Korea, cultural competence of organization, besides global networking could participate on how a medical institute might influence the competitors (14). The ability of health practitioners is the key factor to transfer the positive communication skills and cultural competence to the foreign patients (30, 31) accordingly, in order to provide these communication skills across the cultures, expert training is required, which seems to play a role in promotional policy as well.

Promotional programs through global marketing strategies, which is the second pivotal factor in our result, could become as a part of national and organizational goals; as for Korea, lack of a unique brand can be seen as an initial barriers comparing to the competitors (8, 32). Korean government started to promote medical tourism by offering high quality and IT trend medicine, but it seems not convincing enough to attract foreign patients, since they can find the same quality in some other countries. Hence, two factors of “global marketing strategies” and ‘R & D activities” are required.

Beyond all these themes, is the effective role of government in terms of centralizing the policy making, reconfiguring and action regulations; if fact, nowhere is the effect of government more apparent than in comprehensive policy making. Therefore, in Fig. 2, although each five factors can effect on the development separately, the role of government is shown to be more essential since it can exacerbate the effect of other factor in negative or positive trend as well. The associated Korean authorities could either provide the valid information on global market or encourage the organizations to assign the budget for research and development projects (R & D) (14), improve their competitive advantage and notice the special training for their employees. Other countries, similar to Korea, have included medical tourism in their tourism marketing strategies (25); but the difference in Korea is the shortage of a centralized system for promotion and training. Although Korean government lately tend to provide free training for health practitioners working with foreign patients, there is a need to make sure whether the measures are in right place or not, and whether both organizations and individuals are convinced enough to sustain the competitiveness through innovative services (14).

In this study, only the internal barriers have been investigated, meanwhile, we mentioned to the influence of external factors, particularly the new emerging trend(s) in region. Similarly, this factor has been mentioned as “chance” in the Porter diamond model for the competitiveness of Korea in global healthcare market (14) which implies on the factors that may change the planning and are out of the control. In Korea, the policy making and reconfiguring the goals could not be addressed successfully, unless conducting a research on the issue that what/how competitive advantage are being offered by the competitors. Malaysia, for instance, is a good example of focusing on cultural competence as a competitive advantage through its internal ethnic diversity (4). Moreover, although the participants were all informed and active in current medical tourism situation in Korea, the small number of respondent might led us not to generalize the result, except where it could be comparative with similar studies in other countries. Eventually, conducting the similar research from the demand side perspective would also be beneficial to provide a comparison and to determine their basic needs.

## Conclusion

The result and discussion suggest that South Korea is among the top destinations for medical tourism in terms of high quality infrastructure and medicine designed based on new emerged technologies. Moreover, designing an accurate comprehensive plan, in advance, supports the details. Nevertheless, it appears that number of foreign patients coming to Korea is facing a decreasing trend. The factors identified here are the barriers which hinder the development of medical tourism. To combat these obstacles in Korea, government should reconfigure the policy and planning, especially in terms of promotion. Moreover, the problem of communication skills and training the specialist in medical tourism should be addressed. The key factor among the barriers is the lack of an efficient and centralized government support; Korea does appear to be moving forward in this issue, as indicated by the recent training support and encouraging plan.

## Ethical considerations

Ethical issues (Including plagiarism, informed consent, misconduct, data fabrication and/or falsification, double publication and/or submission, redundancy, etc.) have been completely observed by the authors.
